# Models for the Evolution of GC Content in Asexual Fungi *Candida albicans* and *C. dubliniensis*

**DOI:** 10.1093/gbe/evt170

**Published:** 2013-10-31

**Authors:** Marie-Claude Marsolier-Kergoat

**Affiliations:** ^1^CEA/CNRS/Univ Paris-Sud, FRE 3377, Gif-sur-Yvette, France

**Keywords:** replication, GC content, asexual lineage, *Candida*, mitotic recombination, GC-biased gene conversion

## Abstract

Although guanine–cytosine (GC)-biased gene conversion (gBGC) following meiotic recombination seems the most probable mechanism accounting for large-scale variations in GC content for many eukaryotes, it cannot explain such variations for organisms belonging to ancient asexual lineages, such as the pathogenic fungi *Candida albicans* and *C. dubliniensis*. Analysis of the substitution patterns for these two species reveals a strong anticorrelation between the synonymous transition rates at third codon positions. I propose two models that can account for this observation. According to the first model, the evolution of GC content is driven by gBGC linked to mitotic recombination, either associated with parasexuality or with damage repair. Variations in the GC content thus reflect variations in the strength of gBGC, presumably variations in the mitotic recombination rate. According to the second model, the evolution of GC content is driven by misincorporation errors during the process of DNA replication in S phase. This model proposes that variations in GC content are due to variations in the proportions of dCTPs and dGTPs at the time when sequences are replicated. Experimental data regarding mitotic recombination rates or the variations of dCTPs and dGTPs during S phase are required to validate definitively one of the two models, but in any case, the fit of the models to the data suggests that *C. albicans* and *C. dubliniensis* constitute so far unique examples of GC content evolution driven either by mitotic recombination or replicative errors.

## Introduction

The factors inducing variations in the guanine–cytosine (GC) content, defined as 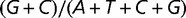 with *A*, *C*, *G,* and *T* representing the frequencies of adenine, cytosine, guanine and thymine, respectively, have long been debated for eukaryotes. Recently, several lines of evidence have accumulated for a mechanism termed GC-biased gene conversion (gBGC), whereby the frequency of meiotic recombination affects the evolution of GC content (reviewed in Eyre-Walker and Hurst [2001] and Duret and Galtier [2009]). Although its molecular details remain unclear, this mechanism relies on the fact that during meiotic recombination, double-strand breaks (DSBs) are repaired through a process involving the formation of DNA heteroduplexes between the strands of the cut and the uncut chromosomes. The mismatches potentially occurring between the two strands of DNA heteroduplexes are repaired, and the whole process tends to favor G or C over A or T bases. Evidence for gBGC has been found in a large range of eukaryotes, including mammals, birds (Duret and Galtier 2009), and the yeast *Saccharomyces cerevisiae* (Birdsell 2002; Mancera et al. 2008; Marsolier-Kergoat 2011), although in the latter case, the recombination rate is more strongly correlated with the present than with the equilibrium GC content (Marsolier-Kergoat and Yeramian 2009).

Although the influence of gBGC seems predominant in sexual eukaryotes, variations in the GC content of species belonging to ancient asexual lineages should not be driven by gBGC associated with meiotic recombination and could therefore show the influence of other factors. The pathogenic yeasts *Candida albicans* and *C. dubliniensis* represent interesting cases in that respect. *Candida albicans* has been shown to exist in a broad range of ploidy states, including haploid, diploid, tetraploid, and aneuploid, which are all mating competent. A diploid–tetraploid parasexual cycle has been demonstrated in *C. albicans*, which includes a switch to the “opaque” physiological state that renders cells mating-competent (Miller and Johnson 2002), conjugation between opaque diploids of opposite mating types to form tetraploids (Hull et al. 2000; Magee and Magee 2000), and subsequent ploidy reduction via a nonmeiotic process termed “concerted chromosome loss,” which generates cells that are diploid or close to diploid (Bennett and Johnson 2003; Forche et al. 2008). A nonmeiotic haploid–diploid parasexual cycle has also been proposed although not formally demonstrated (Hickman et al. 2013). *Candida dubliniensis* is the most closely related species to *C. albicans* described so far. Although it has been much less studied than *C. albicans*, its cycle seems similar and no evidence for meiosis has been found. Mating of *C. dubliniensis* diploids involves opaque switching and fusion between cells of opposite mating types (Pujol et al. 2004). Whether *C. dubliniensis* tetraploids generated by mating revert to diploids by concerted chromosome loss as in *C. albicans* has not been established, but the similarity between *C. dubliniensis* and *C. albicans* cycles, also illustrated by the fact that the two species can mate (Pujol et al. 2004), strongly suggests that *C. dubliniensis* and *C. albicans* belong to asexual lineages at least since their last common ancestor. These two species thus offer a rare opportunity to analyze the mechanisms of genome evolution in the absence of gBGC associated with meiotic recombination.

Interestingly, both *C. albicans* and *C. dubliniensis* genomes exhibit a large heterogeneity in GC content: the GC content of the third codon positions (the GC3 content), when averaged over 15 adjacent genes, shows almost 5-fold variations (between ∼ 0.1 and 0.5), in comparison with less than 2-fold variations in the sexual yeast *S. cerevisiae* (between 0.3 and 0.5; Lynch et al. 2010). Mutation rates in *C. albicans* and *C. dubliniensis* lineages were analyzed using the related species *C. tropicalis* as outgroup. This study revealed a strong anticorrelation between the A:T to G:C and the G:C to A:T transition rates. I propose two models for the evolution of genome composition that could account for this feature.

I first consider the possibility that the main factor responsible for variations in GC content in *C. albicans* and *C. dubliniensis* could be gBGC associated with mitotic recombination. In *C. albicans*, mitotic recombination can be linked either to parasexuality or to the repair of accidental DSBs. Indeed, *C. albicans* diploid–tetraploid parasexual cycle involves extensive genetic recombination between homologous chromosomes in a subset of the progeny, and these recombination events are dependent upon the homolog of Spo11, the endonuclease responsible for the formation of meiotic DSBs in sexual organisms (Forche et al. 2008). Whether mitotic recombination (operating either as a repair mechanism or during parasexual cycles) is associated with gBGC is presently unknown but cannot be excluded. Advancing a model proposed by Duret and Arndt (2008), I show that the hypothesis of gBGC linked to mitotic recombination leads to a theoretical relationship between the A:T to G:C and the G:C to A:T transition rates that is compatible with the observations. If the model is correct, mitotic recombination in *C. albicans* and in *C. dubliniensis*, as revealed by its associated gBGC, would appear to differ from meiotic recombination by a number of features, in particular by the fact that DSBs would not be preferentially located in intergenes, and would not exhibit a higher frequency on small chromosomes and a lower frequency around centromeres.

I then consider the hypothesis that the GC content in *C. albicans* and *C. dubliniensis* could reflect variations in mutational biases linked to replication. This mechanism was first suggested by Wolfe et al. (1989) and was based on three observations: 1) the pattern of base misincorporation by DNA polymerases is affected by deoxynucleoside triphosphate (dNTP) concentrations (e.g., the base G will be preferentially misincorporated into DNA if replication occurs in the presence of a dGTP-rich pool of dNTPs) (e.g., Fersht 1979), 2) the relative concentrations of dNTPs can vary during S phase (e.g., Leeds et al. 1985), and 3) in many cells, DNA replication follows a spatiotemporal program whereby parts of the genome are systematically replicated either at the beginning, in the middle, or at the end of the S phase. Subsequently, several models of DNA replication were developed to describe the relationships between the total mutation rate of a sequence, its GC content, and the proportion of dCTPs and dGTPs in the pool of dNTPs at the time it is replicated (Wolfe 1991; Eyre-Walker 1992; Gu and Li 1994). These few studies on the potential impact of replication on the GC content have to be distinguished from a large body of literature that has analyzed the influence of replication on the GC and the TA skews, defined as 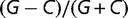 and 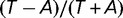, respectively, originally in bacteria (Lobry 1996) and more recently in eukaryotes (Touchon et al. 2005; Arneodo et al. 2011), including yeast species (Agier and Fischer 2012; Marsolier-Kergoat and Goldar 2012). These latter analyses have shown how variations in GC and TA skews along chromosomes can arise from differences in the mutation or the repair rates of the leading and the lagging strands. However, the GC content is independent of the GC and TA skews, and differences between the leading and the lagging strands cannot account for variations in the GC content.

I develop a model for GC content evolution driven by replicative errors that accounts for the observed anticorrelation between the A:T to G:C and the G:C to A:T transition rates. According to this model, the transition rates of a given sequence are directly linked to the proportion 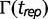 of dCTPs and dGTPs in the pool of dNTPs at the time *t_rep_* when this sequence is replicated during the S phase. The average variations of 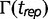 can therefore be extracted from substitution rates and replication timing data. When the data of *C. albicans* and *C. dubliniensis* are analyzed in the frame of this model, the inferred values of 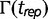 show large variations during the S phase and appear to have globally increased before the divergence of *C. albicans* and *C. dubliniensis* lineages.

Neither of the two models presented here can be definitively validated using the available experimental evidence, but the issue could be settled by the determination of mitotic recombination rates or of the proportions of dCTPs and dGTPs during the S phase.

## Materials and Methods

### Sequence Data

The sequences of all open reading frames (ORFs) without introns of *C. albicans* strain SC5314, of *C. dubliniensis* strain CD36, and of *C. tropicalis* strain ATCC MYA-3404 were downloaded from the Candida Genome Database web pages http://www.candidagenome.org/download/sequence/C_albicans_SC5314/Assembly21/current/ (version of November 2011, last accessed November 19, 2013), http://www.candidagenome.org/download/sequence/C_dubliniensis_CD36/current/ (version of June 2010, last accessed November 19, 2013) and http://www.candidagenome.org/download/sequence/C_tropicalis_MYA-3404/current/ (version of June 2010, last accessed November 19, 2013), respectively.

The ortholog mappings among *Candida* species were downloaded from the Candida Genome Database web page http://www.candidagenome.org/download/homology/orthologs/All_Species_Orthologs_from_CGOB.txt (version of June 2012, last accessed November 19, 2013). These mappings are derived from the curated syntenic groupings at the Candida Gene Order Browser (http://cgob3.ucd.ie/ [last accessed November 19, 2013], Fitzpatrick et al. 2010).

For the analysis of intergenic GC content in *C. albicans* and *C. dubliniensis*, only sequences between annotated chromosomal features were taken into account. The files containing these sequences were downloaded from the Candida Genome Database web pages http://www.candidagenome.org/download/sequence/C_albicans_SC5314/Assembly21/current/ and http://www.candidagenome.org/download/sequence/C_dubliniensis_CD36/current/ (last accessed November 19, 2013), respectively.

### Analysis of Substitution Patterns

A total number of 5,249 sets of orthologous ORFs were analyzed. Multiple sequence alignments guided by amino acid translations were performed using the TranslatorX software (downloaded from http://www.translatorx.co.uk/ [last accessed November 19, 2013], Abascal et al. 2010).

The substitutions having occurred in *C. albicans* and *C. dubliniensis* lineages since their divergence were estimated using *C. tropicalis* as an outgroup to infer the ancestral nucleotide sequences, using parsimony. The analysis was restricted to the codons whose first two bases are identical in all three species. When the alignments showed that the third codon base was identical in *C. albicans* and *C. dubliniensis* sequences, this base was considered to correspond to the ancestral sequence. When the third codon base was found to differ in *C. albicans* and *C. dubliniensis*, the ancestral base was considered to be the one occurring in *C. tropicalis*, if it was identical to the one of either *C. albicans* or *C. dubliniensis* sequence. Sites where the bases of *C. albicans*, *C. dubliniensis,* and *C. tropicalis* are all different were disregarded. No correction for multiple base substitution was attempted. For the analysis of synonymous transitions, the set of codons considered was further restricted to codons different from ATG, ATA, TGA, and TGG in *C. albicans* sequence.

The substitution rates were estimated by dividing the number of inferred substitutions by the number of inferred, potentially mutable, ancestral sites. The substitution rates for third codon positions corresponding to mutations occurring in the *C. albicans* lineage were computed on nonoverlapping 10-kb windows defined for the *C. albicans* genome and representing 1,424 sets of codons. For simplicity of comparison, the same sets of codons were used to compute the substitution rates corresponding to mutations occurring in the *C. dubliniensis* lineage.

### Computational and Statistical Analyses

Data sets were produced and analyzed with custom Python scripts. Statistical analyses were performed with the R environment (R Development Core Team 2008).

Approximations of the values of 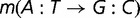, 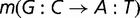 (the mutation rates corresponding to the transitions from A:T to G:C and from G:C to A:T, respectively) and *f* (the fraction of recombination hotspots in DNA sequences) for the model of gBGC linked to mitotic recombination were found as follows. Denoting by 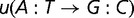 and 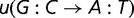 the substitution rates for the transitions from A:T to G:C and from G:C to A:T, respectively, two sets of genomic fragments for which the values of the strength *s* of gBGC are close to 0 were defined: the set corresponding to the 10% of the genomic fragments with the lowest values of 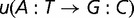 and the set corresponding to the 10% of the genomic fragments with the highest values of 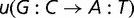. The approximations for 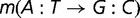 and for 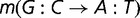 were taken as the mean of the two average values of 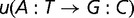 and of 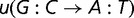, respectively, determined for each set. An estimate of the value of *f* was found by taking the set corresponding to the 10% of the genomic fragments with the highest values of 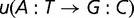, considering that the average value of 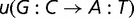 for the genomic fragments of this set approximates 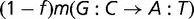. Estimates were also computed using sets including either 5% or 20% of the genomic fragments, instead of 10%, with similar results. Here are the values obtained (the first one corresponds to 5% of the genomic fragments and the second one to 20%): for *C. albicans*, 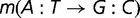: 0.027–0.036, 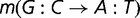: 0.18-0.15, and *f*: 0.64–0.51; for *C. dubliniensis*, 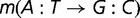: 0.0093–0.021, 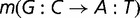: 0.32–0.23, and *f*: 0.78–0.65.

The R package segmented (Muggeo 2008) was used to fit the relation between Γ and the replication time *t_rep_* with a regression model with a broken-line relationship. The replication data for *C. albicans* were taken from Koren et al. (2010). The original values of replication timing (GSE17963_final_data.txt) were scaled between 0 and 1, corresponding to the beginning and the end of the S phase, respectively.

## Results

### Substitution Patterns in *C. albicans* and *C. dubliniensis*

Because mutational processes are investigated, only sequences undergoing the weakest selective pressure can valuably be taken into account. For the *Candida* genomes in which introns are scarce, the study is limited to third codon positions and to intergenes. Moreover, because the intergenic regions of *C. albicans* and of *C. dubliniensis* cannot be easily aligned, substitution patterns can only be established for third codon positions. This was done as described in Materials and Methods, using parsimony and *C. tropicalis* as outgroup to infer the ancestral sequence. 

Among the 1,661,332 positions analyzed, 141,935 and 147,492 sites have undergone substitution in the lineages of *C. albicans* and of *C. dubliniensis*, respectively, since their divergence. I first examined synonymous transitions, taking into account all codons to the exception of ATG, ATA, TGA, and TGG. Let 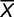 denote the base complementary to *X* and 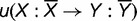 be the substitution rate from 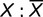 to 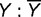. The estimates of the rates were similar in the *C. albicans* and *C. dubliniensis* lineages: 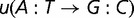 was found equal to 0.062 and 0.063 and 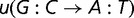 equal to 0.090 and 0.097, respectively, for *C. albicans* and for *C. dubliniensis*.

**Fig. 1.—:**
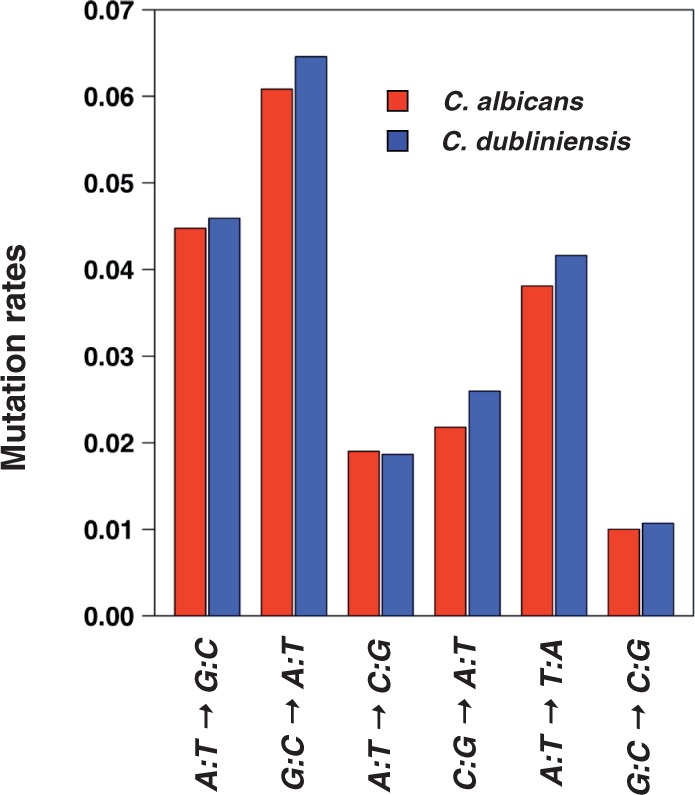
Mutation rates at third codon positions for 4-fold degenerate codons in the lineages of *C. albicans* and *C. dubliniensis*.

The analysis was then restricted to 4-fold degenerate codons to study synonymous transversion rates. The number of positions examined was reduced by a factor of 3 to 506,945, with 50,806 and 53,391 substitutions occurring in the lineages of *C. albicans* and of *C. dubliniensis*, respectively. Figure 1 shows that the substitution rates are similar in the two lineages and that even among 4-fold degenerate codons, the transition rates are higher than transversion rates, as it is usually the case.

**Fig. 2.—:**
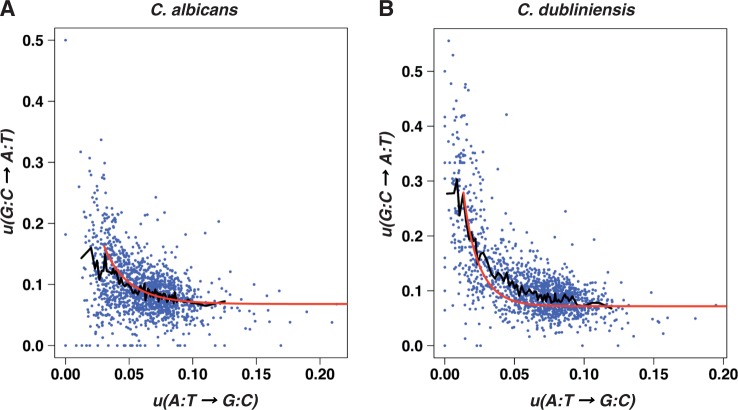
Variations of the transition rate 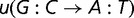 as a function of the transition rate 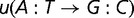 in the lineages of *C. albicans* (*A*) and *C. dubliniensis* (*B*). The red lines represent the theoretical curves corresponding to equations (4) and (5) for the estimates of the parameters given in the main text. The black lines correspond to moving average values that were computed for bins of 20 points.

Relationships between substitution rates were analyzed by dividing the *C. albicans* genome into 1,424 nonoverlapping 10-kb windows for which the rates were calculated. When the synonymous transition rates were computed for all codons except ATG, ATA, TGA, and TGG, a strong anticorrelation was observed between 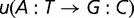 and 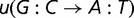 both in *C. albicans* (Spearman correlation coefficient 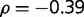, 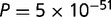, fig. 2*A*) and *C. dubliniensis* lineages (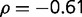, 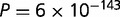, fig. 2*B*). This anticorrelation between 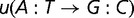 and 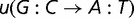 was also observed when the transition rates were computed for the set of 4-fold degenerate codons (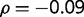, 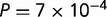 and 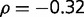, 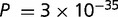, respectively, for *C. albicans* and *C. dubliniensis* lineages).

Similar anticorrelations between synonymous transversion rates could have been expected for the set of 4-fold degenerate codons but were not observed. Thus, 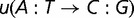 and 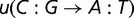 are positively correlated in the *C. albicans* lineage (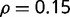, 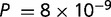) and are insignificantly anticorrelated in the *C. dubliniensis* lineage (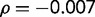, 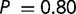). However, the analysis of transversion rates is severely hampered by the low number of transversions observed. For instance, the 10-kb windows include on average 1.4 C:G to A:T substitution occurring in *C. albicans* lineage. Under these conditions, the inconsistent results observed for the correlation between transversion rates in the *C. albicans* and *C. dubliniensis* lineages are difficult to interpret. Alternatively, they could suggest that the mechanisms operating on transition mismatches are different from those operating on transversion mismatches.

**Fig. 3.—:**
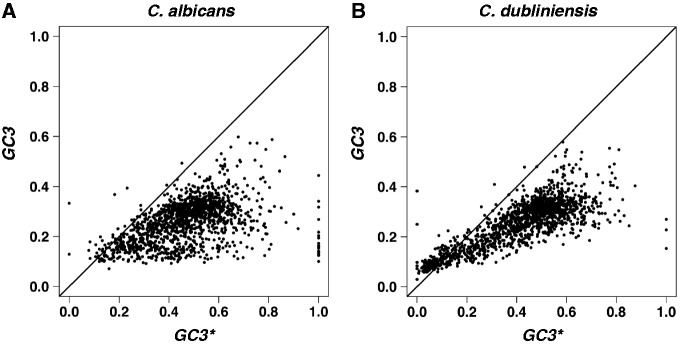
The present GC3 content, *GC3*, is plotted as a function of the equilibrium GC3 content, 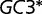, for *C. albicans* (*A*) and for *C. dubliniensis* (*B*). The solid lines correspond to the linear equation 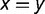.

The equilibrium GC3 content, 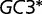, was calculated using the model of Sueoka (1962) as the ratio between the AT to GC substitution rates [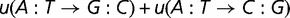] and the sum of the AT to GC and GC to AT substitution rates [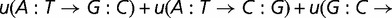 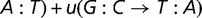]. As shown in figure 3, 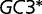 and *GC3* (the current GC3 content) are strongly correlated (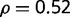, 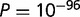 and 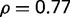, 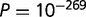, for *C. albicans* and *C. dubliniensis*, respectively). For both species, the GC3 content is far from equilibrium and 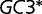 is almost always higher than *GC3*. Similar results were observed when only 4-fold degenerate codons were taken into account (supplementary fig. S1, Supplementary Material online).

As a conclusion, the main characteristic revealed by these substitution analyses is a strong anticorrelation between the synonymous transition rates at third codon positions in the *C. albicans* and *C. dubliniensis* lineages. In the absence of gBGC linked to meiotic recombination, which models could account for this feature? A first hypothesis is that such an anticorrelation might result from selection on codon usage, because selection for the accuracy of translation can affect synonymous codon usage (Akashi 2001). One can imagine for example that the set of preferred codons recently changed and now includes many codons with G or C at the third position. There would be a selective pressure to increase the proportion of these preferred codons in highly translated sequences, which would simultaneously tend to increase 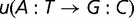 and decrease 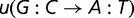. However, the GC3 content and the GC content of intergenes, measured on nonoverlapping 10-kb genome fragments, are highly correlated in both species (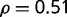, 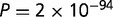 and 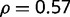, 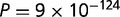 for *C. albicans* and for *C. dubliniensis*, respectively, see supplementary fig. S2, Supplementary Material online), which strongly argues against the hypothesis of selection on codon usage. I have therefore developed two explanatory models, based either on gBGC linked to mitotic recombination or on replication-associated mutational biases.

### A Model for the Evolution of GC Content Driven by gBGC Linked to Mitotic Recombination

The model is based on equations presented by Duret and Arndt (2008) for genome evolution driven by gBGC linked to meiotic recombination, which should also be relevant for mitotic recombination. According to this model, gBGC occurs only in recombination hotspots, whereas all other sequences undergo neutral evolution. We suppose that *f*, the fraction of hotspots in DNA sequences, 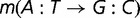 and 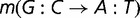, the mutation rates corresponding to the transitions from A:T to G:C and from G:C to A:T, respectively, are constant over a given genome. The substitution rates 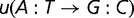 and 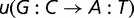 are given by
(1)
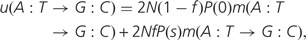

(2)
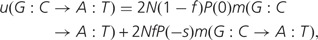

where *N* is the effective population size and *P*(*s*) is the probability that a mutation subject to gBGC of strength *s* will be fixed. The variable *s* changes according to the genomic locus and depends on several parameters including the rate of mitotic recombination, the length of heteroduplex DNA, and the bias in the repair of mismatches. Nagylaki (1983) has shown that gBGC behaves like selection of a semidominant mutation with
(3)
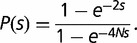

In the absence of gBGC, 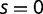 and 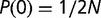, which corresponds to the probability of fixation under random drift. In all cases, we have 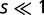 so that 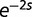 can be approximated by 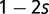 and the equations can be simplified
(4)
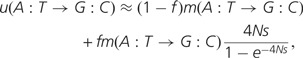

(5)
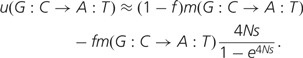

The local transition rates thus vary as a function of the strength *s* of gBGC in the following way: as the value of *s* increases from 0, 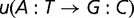 increases from 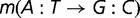, and 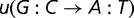 decreases from 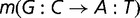 to 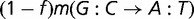. As described in Materials and Methods, I found estimates of 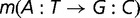 equal to 0.030 and 0.013, estimates of 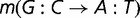 equal to 0.16 and 0.28, and estimates of *f* equal to 0.58 and 0.74 for *C. albicans* and for *C. dubliniensis*, respectively. The red lines in figure 2 show the theoretical curves corresponding to equations (4) and (5) for 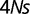 varying between 0 and 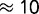 for *C. albicans* and for 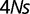 varying between 0 and 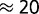 for *C. dubliniensis*, with the estimates of the parameters given above. The curves are close to the black, moving average lines. However, the goodness of fit of the model to the data cannot be rigorously assessed because the model cannot account for several data points [e.g., all the data points such that 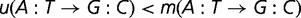].

Let’s now consider what information on mitotic recombination can be derived from this model if relevant to the two *Candida* species. It must be noticed that 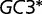 is largely determined by *Ns*. Indeed, transitions represent a large fraction of the substitutions that have changed the GC3 content in *C. albicans* and *C. dubliniensis* lineages since their divergence (0.90 in both cases). Let’s define *R_t_* as the following ratio between the transition rates
(6)
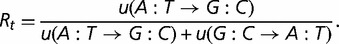

*R_t_* is approximately equal to 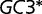: 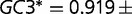 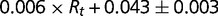 and 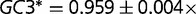 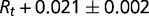, for *C. albicans* and for *C. dubliniensis*, respectively. Because *R_t_* is an increasing function of *Ns*, the observation that 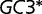 is almost always higher than *GC3* for both *C. albicans* and *C. dubliniensis* (fig. 3) could be explained either by changes in *N* or by an increase in *s* over the whole genome, before the divergence of *C. albicans* and *C. dubliniensis* lineages.

If we further suppose that, for a given genome, variations of 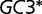 reflect variations in the rate of mitotic recombination, we can compare some characteristics of mitotic recombination, as revealed by the variations of 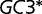, with the usual features of meiotic recombination. First, in a wide variety of eukaryotes, the rate of meiotic recombination is higher in smaller chromosomes due to the requirement of at least one crossover per chromosome (or per chromosome arm) per meiosis. This results in a strong anticorrelation between the average 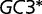 of a chromosome and its length (reviewed in Duret and Galtier [2009]; my unpublished data for *S. cerevisiae*). By contrast, chromosome length and average 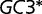 are weakly correlated in *C. albicans* and *C. dubliniensis* (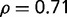, 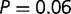 and 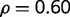, 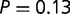, respectively). This observation can be explained by the facts 1) that recombination between homologous chromosomes is not required for the completion of *C. albicans* parasexual cycle (Forche et al. 2008) and 2) that the number of mitotic recombination events linked to the repair of accidental DSBs is expected to be constant per sequence length at large scale. Second, meiotic recombination is usually suppressed near centromeres (Choo 1998), which results in centromeres being located in GC-poor troughs (presumably caused by a reduction of gBGC) in all the yeast species that were recently examined, except in *C. albicans* and *C. dubliniensis* (Lynch et al. 2010). This suggests that mitotic recombination is not inhibited near centromeres in these latter species. Finally, in *S. cerevisiae* and in other sexual fungi, meiotic DSBs are preferentially located in intergenes, which has been suggested to lead to higher values of *GC3* at the 5′ and 3′ ends of genes (Marsolier-Kergoat 2011). The fact that *GC3* does not increase at the ends of the genes in *C. albicans* and *C. dubliniensis* (Marsolier-Kergoat 2011 and my unpublished results) indicates that mitotic DSBs are not preferentially located in intergenes in these organisms.

In summary, a model of GC content evolution driven by gBGC linked to mitotic recombination, either associated with parasexuality or with damage repair, can account for the strong anticorrelation between 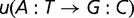 and 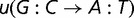 observed in *C. albicans* and *C. dubliniensis*. If this model is correct, then variations of *GC3* reflect variations in the strength *s* of gBGC, presumably variations in the mitotic recombination rate. The validity of the model could thus be tested experimentally by determining the mitotic recombination rates along the chromosomes. We will now describe another model, based on replication, that could also explain our observations.

### A Model for the Evolution of GC Content Driven by Replication Errors

Let’s consider a DNA polymerase *E*, which has already extended a primer DNA up to position *n*. Following Fersht (1979) and Gu and Li (1994), we will model the insertion of the following nucleotide at position *n*+1 as a Michaelis–Menten process. We have
(7)
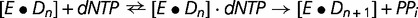

with 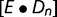 corresponding to the polymerase–DNA complex and *PP_i_* to pyrophosphate. This model is supported by in vivo and in vitro experiments showing that the dependence of dNTP incorporation on dNTP concentration conforms to a Michaelis–Menten equation (e.g., Dresler et al. 1988). Given that *X* is the correct nucleotide for position *n*+1, let *v*(*X*), 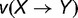, 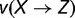, and 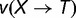 be, respectively, the rate of correct incorporation and the rates of incorrect incorporation of nucleotides *Y*, *Z,* and *T* instead of *X*. Nucleotides *Y*, *Z,* and *T* are distinguished by the fact that the 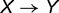 substitution corresponds to a transition, whereas the 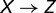 and 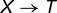 substitutions correspond to transversions. The rates *v* are determined by the following Michaelis–Menten equations
(8)
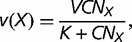

(9)
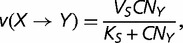

(10)
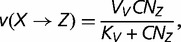

(11)
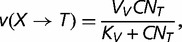

where *V*, *V_S_*, and *V_V_* are the maximum rates of polymerization for correct and incorrect nucleotides, corresponding either to transitions (*V_S_*) or to transversions (*V_V_*), *K*, *K_S_*, and *K_V_* are the Michaelis constants for correct and incorrect polymerization, corresponding either to transitions (*K_S_*) or to transversions (*K_V_*), *C* is the total concentration of dNTPs, and *N_X_* is the proportion of a given dXTP among the pool of dNTPs. Because it is generally observed that 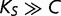 and 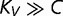 (e.g., Wong et al. 1991), equations (9)–(11) can be simplified so that we get
(12)
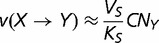

and similar equations for 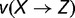 and 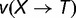. Let 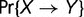 be the probability that the incorrect nucleotide *Y* is incorporated instead of the correct nucleotide *X*.
(13)
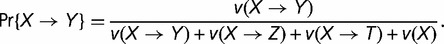

(14)
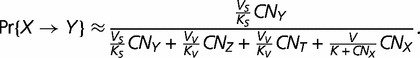

After some algebra, we have
(15)
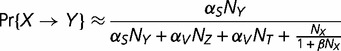

with 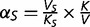, 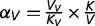, and 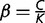. Equation (15) is comparable to the equation derived by Gu and Li (1994) for 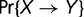. Noting that the magnitude of 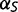 and 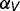, which correspond to discrimination coefficients against incorrect nucleotides, is less than 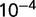 (Echols and Goodman 1991), and assuming that the value of *β* in eukaryotic cells usually lies in the range of 30–100 that can be estimated from data on human fibroblasts (Dresler et al. 1988), we can write
(16)
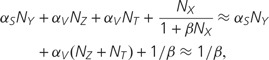

which allows to drastically simplify the expression for 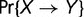

(17)
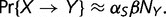

Let’s now consider the replication of a double-stranded DNA fragment occurring at time *t_rep_* during S phase. Both strands of DNA are submitted to replicational errors, and the primary event leading to a change from 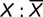 to 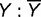 could be either the misincorporation of *Y* instead of *X* or the misincorporation of 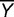 instead of 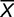. Accordingly, 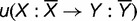 corresponds to the sum of the probabilities that *Y* is misincorporated instead of *X* and that 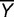 is misincorporated instead of 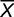, multiplied by the probability that these misincorporations are not corrected and that the corresponding mutations become fixed in the population. We will suppose that the global probability of noncorrection and of fixation, *p_nc_*, is a constant.
(18)
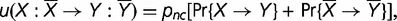

(19)
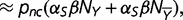

(20)
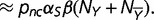

Let 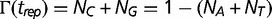 be the proportion of dCTPs and dGTPs in the pool of dNTPs at time *t_rep_* when the sequence is replicated. The transition rates can be expressed as
(21)
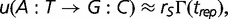

(22)
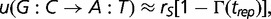

where 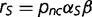.

These equations describe substitution rates corresponding to evolutionary time under the conditions that the replication timing program is stable (genomic sequences are always replicated in the same order) and that the variations of Γ as a function of *t_rep_* are conserved so that a given sequence is always replicated in the presence of the same proportion of dCTPs and dGTPs in the pool of dNTPs. Conservation of the replication timing program was recently demonstrated in related budding yeast species (Müller and Nieduszynski 2012), which makes the first of these assumptions plausible.

From equations (21) and (22), we can deduce a simple relation between the transition rates
(23)
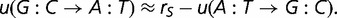

**Fig. 4.—:**
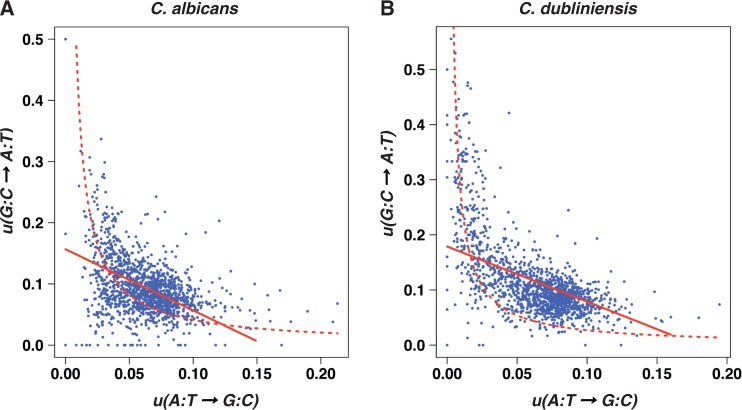
Variations of 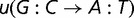 as a function of 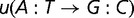 in the lineages of *C. albicans* (*A*) and *C. dubliniensis* (*B*). The solid red lines represent the fitting curves of equation 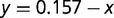 and 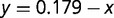 in (*A*) and (*B*), respectively. The dotted red lines correspond to the fitting curves of equations 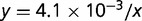 and 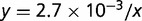 in (*A*) and (*B*), respectively.

The model thus accounts for the anticorrelation observed between 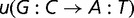 and 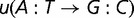 in *C. albicans* and *C. dubliniensis* lineages. The solid lines in figure 4 represent the lines of equation 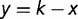 with the value of *k* determined as giving the best fit to the data (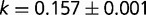 and 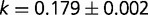, which corresponds to residual standard errors equal to 0.042 and 0.058, for *C. albicans* and for *C. dubliniensis*, respectively). However, it can be noticed that the model does not account for the shape of the curve for the lowest values of 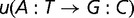, especially for *C. dubliniensis*. I therefore examined whether another model could better explain this trend.

Several authors (Bernardi and Ninio 1978; Wolfe 1991; Eyre-Walker 1992) have developed a simpler model for misincorporation errors occurring during DNA replication according to which
(24)
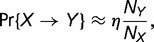

where *η* is a constant. Using the same reasoning and the same notations as above we have for a double-stranded DNA fragment
(25)
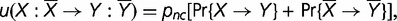

(26)
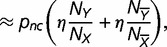

(27)
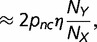

if we assume that 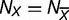 for all dXTPs. Regarding the transition rates, we get
(28)
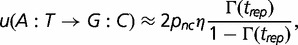

(29)
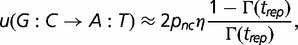

so that, in this case also, we can deduce a simple relationship between the transition rates
(30)
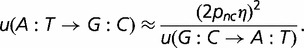

The dotted lines in figure 4 correspond to the lines of equation 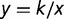 with the value of *k* determined as giving the best fit to the data (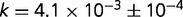 and 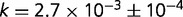 for *C. albicans* and for *C. dubliniensis*, respectively). These curves capture the initial downward trend of the data better than the straight lines previously determined, but on the whole this model fits the data less well than the linear one (residual standard errors equal to 0.051 and 0.079 for *C. albicans* and *C. dubliniensis*, respectively).

I also attempted to introduce proofreading mechanisms into these replication models. I considered the simplest possible kinetic model of proofreading proposed by Bernardi and Ninio (1978), according to which the probability that proofreading does not occur depends on the extension rate of the polymerase from the upstream mismatched nucleotide and is proportional to *N_X_*/(*K*+*N_X_*), where *K* is a constant and *N_X_* represents the proportion of the correct dNTP for the site downstream. However, the introduction of proofreading complicates the expression of 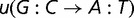 and 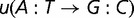 to such a point that no testable relationship between 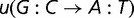 and 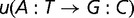 can be derived so that the model can no longer be validated by experimental data. In the following, I will therefore consider equations (21) and (22) as giving the best testable approximations of 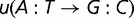 and 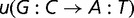 corresponding to a model of GC content evolution driven by replication errors.

We will now examine what information can be extracted from genomic data relative to *C. albicans* and *C. dubliniensis* in the frame of this model, starting with the variations of the proportion of dCTPs and dGTPs during S phase. From equations (21) and (22), we can deduce the equality between 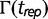, the proportion of dCTPs and dGTPs at *t_rep_*, and the ratio *R_t_* previously defined
(31)
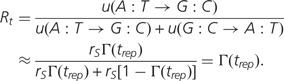

If the spatiotemporal program of DNA replication has remained globally unchanged in the *C. albicans* lineage since its divergence from *C. dubliniensis*, then this program associates all genomic loci with their average replication times *t_rep_*. In the frame of this model, temporal variations of 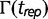 translate into spatial variations of transition rates, and, reciprocally, the variations of 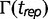 can be inferred from the analysis of local transition rates, knowing the current program of DNA replication.

**Fig. 5.—:**
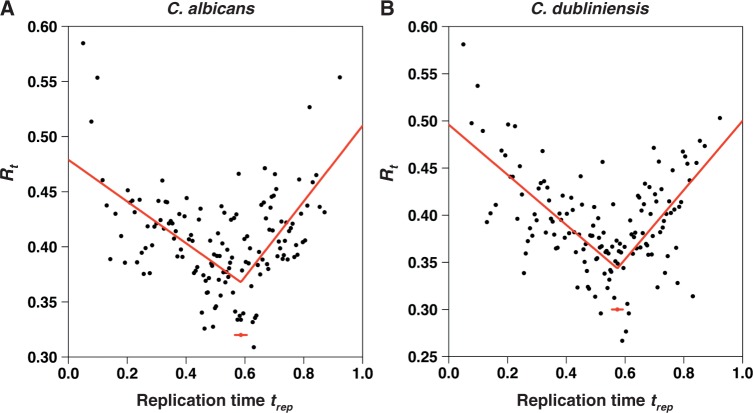
Variations of the ratio *R_t_* of the rates of transitions occurring in the lineages of *C. albicans* (*A*) and of *C. dubliniensis* (*B*). The replication time *t_rep_* is scaled between 0 and 1, which correspond to the beginning and the end of the S phase, respectively. The estimated positions of the breakpoints and the corresponding standard errors are indicated by red dots and segments, respectively. The segmented red lines are the fitting curves described in the main text.

I took advantage of the recent determination of the replication timing profiles in *C. albicans* (Koren et al. 2010) to analyze the variations of 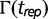. The *C. albicans* genome was split into nonoverlapping 1-kb windows. These 1-kb genome fragments were ranked by their replication times and pooled into bins of 100 kb to compute their average transition rates in the *C. albicans* lineage. The variations of *R_t_* shown in figure 5*A* indicate that 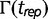 is high at the beginning of the S phase (when *t_rep_* is close to 0), decreases till the middle of the S phase, and increases again in the second part of the S phase. The data can be fitted by a piecewise regression model with two straight segments connected by a breakpoint at 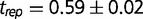 (Pearson correlation coefficient 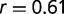). The difference in slope between the two segments is highly significant (Davies’ test, 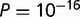).

Under the assumption that the replication programs in *C. albicans* and *C. dubliniensis* lineages have remained similar after their divergence (i.e., considering that orthologous genes have the same *t_rep_* in the two species), comparable variations are observed when plotting the *R_t_* ratio of transitions occurring in the *C. dubliniensis* lineage as a function of *t_rep_* (fig. 5*B*). The breakpoint of the fitting curve (Pearson correlation coefficient 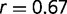) is located at 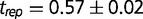, and the difference in slope between the two segments is again highly significant (Davies’ test, 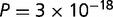).

Let’s now consider how the evolution of the GC content in *C. albicans* and *C. dubliniensis* can be interpreted in the frame of this model. As previously mentioned, transitions represent the large majority of the substitutions that have changed the GC3 content in *C. albicans* and *C. dubliniensis* lineages. We thus have
(32)
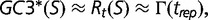

that is, the value of 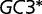 for a given sequence *S* replicated at *t_rep_* is approximately equal to the proportion of dCTPs and dGTPs available at that time.

Accordingly, the fact that for both species, 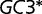 is almost always higher than *GC3* (fig. 3) suggests a global increase in Γ throughout the whole duration of the S phase. This change in the relative concentrations of dCTP and dGTP should have taken place before the divergence of *C. albicans* and *C. dubliniensis* lineages, because 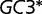 is higher than *GC3* in both lineages. What induces variations in dCTP and dGTP concentrations during the S phase or what could globally increase these relative concentrations throughout the whole duration of the S phase remains largely unknown. However, it is tempting to consider these changes as by-products of nucleotide metabolism without a selective value specifically linked to DNA polymerase misincorporation rates.

As a conclusion, we have seen that a model for the evolution of GC content driven by replication errors can account for the anticorrelation observed between 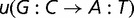 and 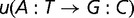 in *C. albicans* and in *C. dubliniensis*. According to this model, local variations of *GC3* are linked to temporal variations (averaged over evolutionary time) of 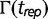, the proportion of dCTPs and dGTPs at time *t_rep_* when the sequences are replicated (considering that the replication timing program and the variations of the relative concentrations of dNTPs as functions of *t_rep_* have been globally stable). The model predicts large variations of 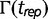 during the S phase, between 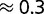 and 0.6, in *C. albicans* and *C. dubliniensis* lineages. If the replication mechanisms that are responsible for the variations of *GC3* in these species still operate, then we should expect these variations of 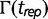 to be conserved, which could be tested experimentally.

## Discussion

The fact that *C. albicans* and *C. dubliniensis* most probably belong to asexual lineages, since at least their last common ancestor provides us with the rare opportunity to study the evolution of GC content in the absence of gBGC linked to meiotic recombination, which is the major driving force of GC content evolution in sexual organisms. The analysis of substitution patterns in *C. albicans* and *C. dubliniensis* lineages reveals a strong anticorrelation between the transition rates 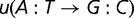 and 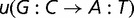. Two models that can account for this observation are proposed. The first model (the “recombination model”) is based on gBGC linked to mitotic recombination, either associated with parasexuality or with damage repair. According to this model, variations in the strength of gBGC, probably due to variations in mitotic recombination rates, induce variations in *GC3*. The second model (the “replication model”) is based on misincorporation errors occurring during DNA replication and proposes that variations in *GC3* are due to variations in the proportions of dCTPs and dGTPs at the time when sequences are replicated.

These two models account for the observed anticorrelation between transition rates but could also have explained anticorrelations between synonymous transversion rates if the latter had been found. Indeed, regarding the recombination model, equations similar to (4) and (5) can be derived for transversion rates
(33)
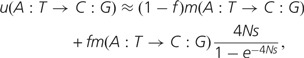

(34)
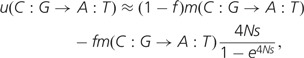

where 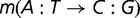 and 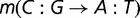 are the mutation rates, considered as constant, corresponding to the transversions from A:T to C:G and from C:G to A:T, respectively. Likewise, for the replication model, we could derive 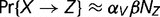 and 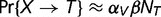 so that we have
(35)
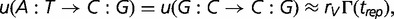

(36)
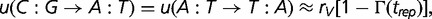

where 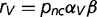, and finally
(37)
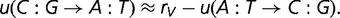

As mentioned earlier, anticorrelations between synonymous transversion rates were neither observed for *C. albicans* nor for *C. dubliniensis*. This result could be due to differences in the mechanisms processing transition and transversion mismatches. For example, the presence of transversion mismatches in heteroduplex DNA could influence the choice of the template strand for DNA repair during mitotic recombination. One can also imagine that the proofreading of transversion mismatches is so efficient during the S phase that the majority of transversions originate from DNA lesions unrelated to replication. In any case, because transitions represent 90% of the substitutions changing the GC3 content in the *C. albicans* and *C. dubliniensis* lineages, the evolution of *GC3* in these lineages has been essentially driven by the mechanisms controlling the transition rates.

It has to be noted that the two models are not mutually exclusive and that replication and mitotic recombination may both influence the GC content of the *Candida* genomes. These two processes affect different parameters in the proposed models. On the one hand, replication-linked mutational biases affect the mutation rates 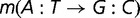 and 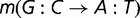, which are supposed to be constant in the recombination model. On the other hand, gBGC affects the probability of allele fixation, whereas the global probability *p_nc_* of error noncorrection and allele fixation is supposed to be constant in the replication model. The effects of replication and mitotic recombination on GC content could thus locally interfere and reinforce or cancel out each other.

It is also worth noting that the replication and the mitotic recombination models could a priori apply to all eukaryotes and that replication and mitotic recombination could combine their effects on GC content with those of gBGC linked to meiotic recombination. A direct way to test the relevance of the replication model for a given organism consists in analyzing the correlation between the proportion 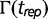 of dCTPs and dGTPs at time *t_rep_* during the S phase and the equilibrium GC content 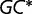 (or the ratio *R_t_* of transition rates) of the sequences replicated at that time. However, data regarding the variations of 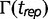 are seldom available. An alternative solution consists in analyzing the variations of 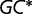 or *R_t_* as a function of *t_rep_*. If a genome exhibits significant variations of GC content that are influenced by replication-associated mutational biases, we expect to observe large variations of *R_t_* as a function of *t_rep_*, with ample trends consistent with slow variations of 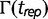 during S phase, as shown in figure 5. Although such a pattern of variation does not constitute a definitive proof in itself, it is at least consistent with the existence of replication-associated mutational biases. By contrast, fluctuations of 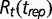 around a time-independent average value indicate that variations of GC content are not influenced by replication-associated mutational biases. This absence of variations at large scale for 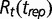 is observed in the case of *S. cerevisiae* (supplementary fig. S3, Supplementary Material online), which suggests that in this organism replication-associated mutational biases do not exist or that the proportion 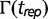 of dCTPs and dGTPs is constant during S phase.

In theory, mitotic recombination rates could also influence GC content through gBGC in sexual eukaryotes. However, for the 82 simple conversion tracts associated with spontaneous mitotic recombination events that were recently analyzed on the right arm of chromosome IV in *S. cerevisiae* (St Charles and Petes 2013), I found no significant increase in the GC content of the converted sequences (data not shown). Accordingly, in the case of *S. cerevisiae,* at least, mitotic recombination events linked to the repair of DNA lesions may not be associated with gBGC.

Regarding the *Candida* genomes, we cannot presently estimate the relevance of the two models because we lack experimental data such as measures of mitotic recombination rates and of the proportion of dCTPs and dGTPs during S phase. Strategies allowing to determine these parameters have already been developed in *C. albicans* or in other species. In particular, mitotic recombination events linked to parasexuality have already been studied in *C. albicans* (Forche et al. 2008), although the number of progeny cells analyzed (13) was too small to give a detailed picture of the spatial variations of the recombination rate. As for the mitotic recombination events linked to the repair of DNA damage, the strategy used for the high-resolution mapping of spontaneous mitotic recombination events in *S. cerevisiae* (St Charles and Petes 2013) could also be used for *Candida* species.

In any case, the validity of any one of the two models for *C. albicans* and *C. dubliniensis* would constitute an original example of GC content evolution. If the recombination model is correct, then we have the first example of GC content evolution driven by gBGC linked to mitotic recombination. The analysis of 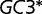 shows that the recombination process associated with gBGC in this case differs from meiotic recombination on several points regarding the distribution of DSBs. If the replication model is correct, then *C. albicans* and *C. dubliniensis* represent the first examples of GC content evolution driven by replicative errors, a mechanism proposed long ago by Wolfe et al. (1989) for which no definite example has so far been found.

## Supplementary Material

Supplementary figures S1–S3 are available at *Genome Biology and Evolution* online (http://www.gbe.oxfordjournals.org/).

Supplementary Data
