# Supplementary material for: Models for the Evolution of GC Content in Asexual Fungi Candida albicans and C. dubliniensis
Source: Genome Biol Evol. 2013 Oct 31;5(11):2205–16. doi: 10.1093/gbe/evt170 (PMC3845650; doi:10.1093/gbe/evt170)
Supplement: Supplementary Data [file supp_5_11_2205__index.html]

Models for the evolution of GC content in the asexual fungi Candida albicans and Candida dubliniensis — Models for the Evolution of GC Content in Asexual Fungi Candida albicans and C. dubliniensis — Supplementary Data 

# Models for the Evolution of GC Content in Asexual Fungi *Candida albicans* and *C. dubliniensis*

## Supplementary Data

files

**Files in this Data Supplement:**

- Supplementary Data - pdf file
- Supplementary Data - pdf file
- Supplementary Data - pdf file
