# Supplementary material for: Models for the Evolution of GC Content in Asexual Fungi Candida albicans and C. dubliniensis
Source: Genome Biol Evol. 2013 Oct 31;5(11):2205–16. doi: 10.1093/gbe/evt170 (PMC3845650; doi:10.1093/gbe/evt170)
Supplement: Supplementary Data [file supp_evt170_Figure_S1.pdf]

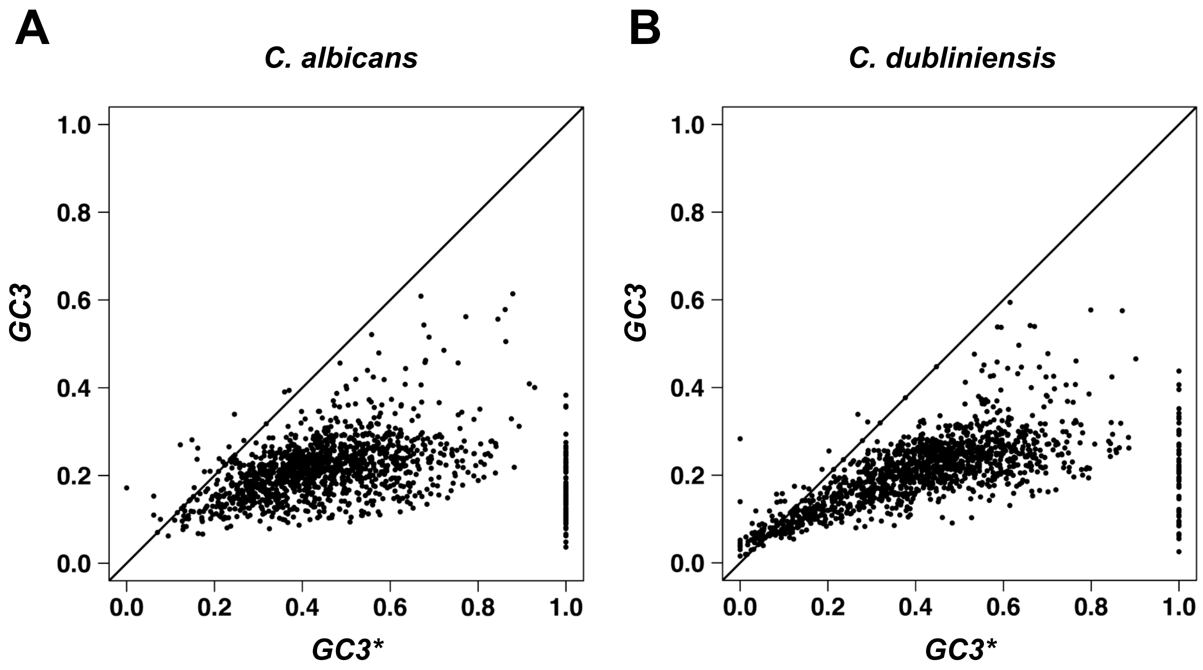

Supplementary Figure S1. The present GC3 content of fourfold degenerate codons, *GC3*, is plotted as a function of their equilibrium GC3 content, *GC3*.* The solid lines correspond to the linear equation $x = y$.
