# Supplementary material for: Models for the Evolution of GC Content in Asexual Fungi Candida albicans and C. dubliniensis
Source: Genome Biol Evol. 2013 Oct 31;5(11):2205–16. doi: 10.1093/gbe/evt170 (PMC3845650; doi:10.1093/gbe/evt170)
Supplement: Supplementary Data [file supp_evt170_Figure_S2.pdf]

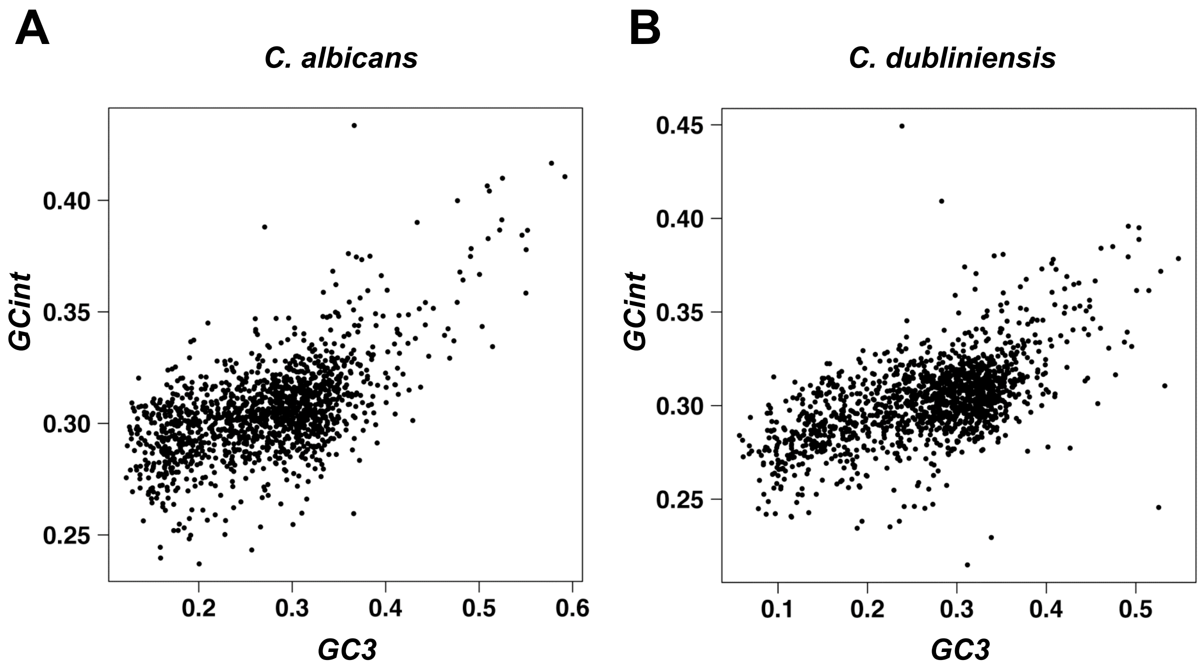

Supplementary Figure S2. The GC content of intergenes, *GCint*, is plotted as a function of the GC3 content, *GC3*, for *Candida albicans* (*A*) and for *Candida dubliniensis* (*B*).
