# Supplementary material for: Models for the Evolution of GC Content in Asexual Fungi Candida albicans and C. dubliniensis
Source: Genome Biol Evol. 2013 Oct 31;5(11):2205–16. doi: 10.1093/gbe/evt170 (PMC3845650; doi:10.1093/gbe/evt170)
Supplement: Supplementary Data [file supp_evt170_Figure_S3.pdf]

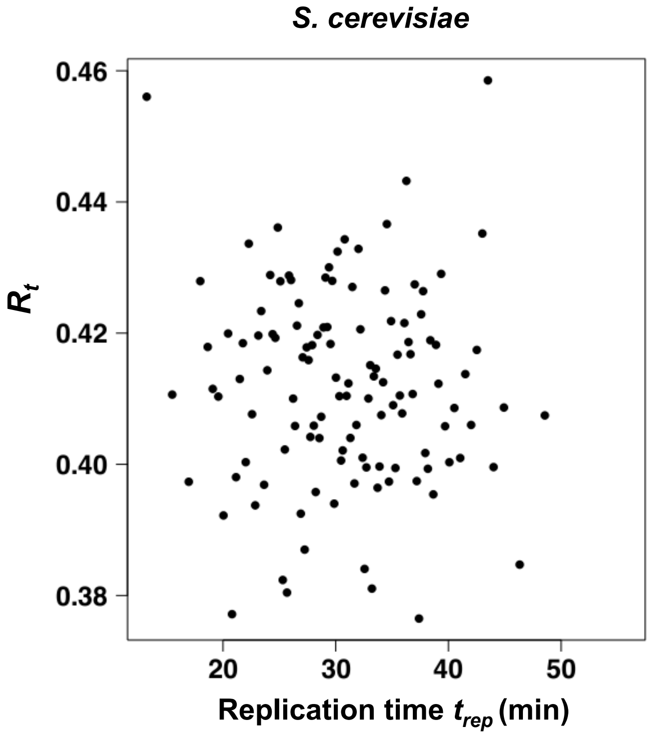

Supplementary Figure S3. Variations of the ratio R_t of the rates of transitions having occurred at third codon positions in the lineage of *Saccharomyces cerevisiae* since its divergence from *Saccharomyces paradoxus*. The sequences of *S. cerevisiae* strain S288C were downloaded from the *Saccharomyces* Genome Database web pages (http:/www.yeastgenome.org), those of *S. paradoxus* and *Saccharomyces mikatae* from the Broad Institute web pages (http:/www.broad.mit.edu/annotation/fungi/comp_yeasts/downloads.html). A total number of 3,967 sets of orthologous open reading frames were analyzed. The substitutions having occurred in the *S. cerevisiae* lineage since its divergence from *S. paradoxus* were estimated using *S. mikatae* as an outgroup to infer the ancestral sequences. Analysis of substitution patterns were performed exactly as described for the *Candida* species in Materials and Methods. Replication timing data were taken from Raghuraman MK et al. 2001. Replication dynamics of the yeast genome. Science 294:115-121. The times are given in minutes after the beginning of S phase. As for the *Candida* species, 1 kb genome fragments were ranked by their replication times and pooled into bins of 100 kb to compute their average transition rates in the *S. cerevisiae* lineage.
